# Precision integrated identification of predictive first-trimester metabolomics signatures for early detection of gestational diabetes mellitus

**DOI:** 10.1186/s12933-025-02978-0

**Published:** 2025-11-14

**Authors:** Sapna Sharma, Yalamanchili Venkata Subrahmanyam, Payal Gupta, Sangeetha Vadivel, Mohan Deepa, Ansh Tandon, Sreekumar Sreedevi, Uma Ram, Priyanka Narad, Dharmeshkumar Parmar, Ranjit Mohan Anjana, Anu Raghunathan, Muthuswamy Balasubramanyam, Viswanathan Mohan, Abhishek Sengupta, Jerzy Adamski, Ponnusamy Saravanan, Venkateswarlu Panchagnula, Dandamudi Usharani, Kuppan Gokulakrishnan

**Affiliations:** 1 Institute of Epidemiology, Research Unit of Molecular Epidemiology, Helmholtz Zentrum München, German Research Center for Environmental Health, 85764 Neuherberg, Germany; 2 CEPD Division, CSIR-National Chemical Laboratory, Dr. Homi Bhabha Road, Pune, 411008 India; 3 Academy of Scientific and Innovative Research (AcSIR), Ghaziabad, 201002 India; 4 Systems Biology and Data Analytics Research Lab, Amity Institute of Biotechnology, Amity University, Uttar Pradesh, Noida, 201313 India; 5 Department of Epidemiology, Madras Diabetes Research Foundation, Chennai, India; 6 Department of Diabetology, Madras Diabetes Research Foundation (ICMR-Collaborating Centre of Excellence) & Dr. Mohan’s Diabetes Specialities Centre (IDF Centre of Excellence in Diabetes Care), Chennai, India; 7 Seethapathi Clinic & Hospital, Chennai, India; 8 Indian Council of Medical Research, Ansari Nagar, New Delhi, India; 9 Department of Cell and Molecular Biology, Madras Diabetes Research Foundation, Chennai, India; 10 Institute of Experimental Genetics, Helmholtz Zentrum München, German Research Center for Environmental Health, Ingolstädter Landstraße 1, 85764 Neuherberg, Germany; 11 Department of Biochemistry, Yong Loo Lin School of Medicine, National University of Singapore, 8 Medical Drive, Singapore, 117597 Singapore; 12 Institute of Biochemistry, Faculty of Medicine, University of Ljubljana, Vrazov Trg 2, 1000 Ljubljana, Slovenia; 13 Populations, Evidence and Technologies, Division of Health Sciences, Warwick Medical School, University of Warwick, Coventry, UK; 14 Department of Diabetes, Endocrinology and Metabolism, George Eliot Hospital, Nuneaton, UK; 15 Centre for Global Health, University of Warwick, Coventry, UK; 16 Department of Food Safety and Analytical Quality Control Laboratory, CSIR-Central Food Technological Research Institute (CFTRI), Mysore, Karnataka 570020 India; 17 Department of Neurochemistry, National Institute of Mental Health and Neuro Sciences (NIMHANS), Hosur Road, Bengaluru, Karnataka 560029 India

**Keywords:** Gestational diabetes mellitus, Metabolomics, Prediction, First trimester, Mass spectrometry, Indian women

## Abstract

**Background and aim:**

Gestational diabetes mellitus (GDM), a common pregnancy-related metabolic disorder, often goes undiagnosed until the second trimester, limiting early intervention opportunities. Given the higher prevalence of GDM in India, there is a critical need to investigate metabolomic biomarkers among Asian Indians, who exhibit greater insulin resistance and are predisposed to developing type 2 diabetes at an earlier age. This study aimed to identify early pregnancy metabolomic signatures predictive of GDM.

**Methods:**

Among 2115 pregnant women from the STratification of Risk of Diabetes in Early pregnancy (STRiDE) study, we performed untargeted metabolomic profiling using UPLC-MS/MS at early pregnancy (< 16 weeks) plasma samples from 100 women—comprising 50 with GDM and 50 normal (without GDM) based on oral glucose tolerance test (OGTT) at 24–28 weeks. Statistical and machine learning approaches, including logistic regression and random forest (RF), were applied to identify GDM-associated metabolites and construct predictive models. Pathway enrichment analysis was conducted using KEGG database annotations.

**Results:**

A total of 49 metabolites were significantly associated with GDM, primarily involving lipid classes such as phosphatidylcholines, sphingomyelins, and triacylglycerols. RF analysis identified a panel of eight metabolites that achieved best predictive performance (AUC 0.880; 95% CI: 0.809–0.951) for GDM. When combined with conventional clinical risk factors, the integrated model showed comparable prediction of GDM with AUC 0.88;: 95% CI: 0.810–0.952). Enrichment analysis highlighted dysregulated pathways including glycerophospholipid and sphingolipid metabolism, autophagy, and insulin resistance.

**Conclusion:**

This study demonstrates the utility of early-pregnancy metabolomic profiling for predicting GDM in Indian women. The eight-metabolite panel offers a promising tool for early risk stratification of GDM, warranting validation in diverse populations.

**Supplementary Information:**

The online version contains supplementary material available at 10.1186/s12933-025-02978-0.

## Research summary

What is currently known about this topic?

- Gestational diabetes mellitus (GDM) remains undiagnosed until the late second trimester, when glucose tolerance tests are routinely administered.
- This late diagnosis limits opportunities for early intervention, emphasizing the need for novel, non-invasive biomarkers to predict GDM earlier in pregnancy.
- Timely identification and management of GDM risk factors are essential to reducing adverse maternal and fetal outcomes.

What is the key research question?

- Are there any metabolomic signatures in early pregnancy (< 16 weeks) that can serve as a predictive marker for identifying GDM.

What is new?

- This study investigates the predictive performance of metabolomic biomarkers in an Indian cohort.
- We identified a panel of eight metabolites during early pregnancy that could predict GDM with high accuracy, underscoring the potential clinical utility of metabolomic signatures in early risk stratification.

How might this study influence clinical practice?

- Integrating the metabolomic biomarker assessment and conventional clinical risk factors could enhance personalized GDM prevention and management at an early stage.

## Introduction

Gestational diabetes mellitus (GDM), defined as glucose intolerance with onset or first recognition during pregnancy, remains one of the most prevalent pregnancy-related metabolic disorders worldwide [1]. The global prevalence of GDM ranges between 5% and 25%, depending on diagnostic criteria and population demographics, and it continues to rise in parallel with increasing rates of obesity and sedentary lifestyles [2–4]. GDM poses considerable risks to both mother and fetus [5], including hypertensive disorders, cesarean delivery, macrosomia [6], neonatal hypoglycemia, and a higher predisposition for type 2 diabetes mellitus (T2DM) [7], and cardiovascular diseases [8–11] later in life for both the mother and offspring. Unfortunately, GDM often remains undiagnosed until the late second trimester, when glucose tolerance tests are routinely administered [12]. This delay limits opportunities for early intervention, underscoring the need for novel, non-invasive biomarkers capable of predicting GDM at earlier stages of pregnancy [13].

Recent studies have highlighted the utility of Mass Spectrometry (MS) -based metabolomics in uncovering potential predictive metabolite biomarkers for GDM [14–16]. A study by Zhao et al. used Liquid Chromatography—Mass Spectrometry (LC-MS) to profile maternal serum in the first trimester and identified a panel of metabolites, including branched-chain amino acids and acylcarnitines, that were significantly associated with subsequent GDM development [17]. Similarly, Scholtens et al. from the Hyperglycemia and Adverse Pregnancy Outcome (HAPO) study explored metabolomic signatures and demonstrated distinct metabolic profiles linked to insulin resistance and glucose dysregulation as early as 10–14 weeks of gestation [18]. Another study by Mokkala et al. reported distinctive metabolic signatures in early pregnancy associated with later GDM onset [19]. Additionally, emerging studies have pointed to disruptions in bile acid metabolism [20–22], aromatic amino acids [23], and microbiome-derived metabolites [24] as key components in the metabolic landscape of GDM. Interestingly, our earlier study has shown that lower telomere length and mitochondrial DNA copy number in early pregnancy were significantly linked to a higher risk of developing GDM later in pregnancy [25]. These findings support the notion that metabolic perturbations precede clinical GDM diagnosis and can be leveraged for early risk prediction.

Lipidomics studies have also shown promise in the context of GDM [26, 27]. Among the most promising metabolite classes, small molecular lipids such as sphingolipids, glycerophospholipids, and fatty acids were shown to play a crucial role in metabolic regulation and disease pathogenesis [28–32]. These molecules hold significant potential as early biomarkers for predicting the onset of GDM, opening new avenues for timely diagnosis and improved maternal and fetal outcomes. A study by Liu et al. identified alterations in phospholipids and sphingolipids in early pregnancy serum samples from women who later developed GDM, indicating potential roles for altered lipid metabolism in GDM pathogenesis [29]. Despite these advancements, there remains a lack of consensus on standardized metabolomic biomarkers for GDM prediction, partly due to heterogeneity in study design, population differences, and analytical platforms. Studies predominantly from European and East Asian cohorts have reported early lipid metabolic alterations associated with GDM, while the data from South Asian populations remain limited despite their high GDM burden. Our study advances this field by aiming to identify a multimetabolite panel in an Indian cohort that not only reflects population-specific metabolic features but also aligns with global metabolomic pathway disturbances linked to GDM. Such cross-ethnic consistency underscores the potential translational value of metabolite panels that capture shared metabolic mechanisms while accounting for population variability.

We therefore, aim to identify early pregnancy (< 16 weeks) metabolomic signatures for GDM in a longitudinal Indian pregnancy cohort from the STratification of Risk of Diabetes in Early Pregnancy (STRiDE) study [33] using comprehensive mass spectrometry profiling. By leveraging the precision of modern MS techniques, applying robust bioinformatic analysis and integrating conventional maternal risk factors, we strive to advance early prediction and risk stratification, which is expected to result in personalized prenatal care and better maternal-fetal outcomes.

## Method

### Study participants

The study population comprised 2115 pregnant Indian women enrolled in the STRiDE study, a prospective, longitudinal cohort that recruited participants at their first antenatal visit, prior to 16 weeks of gestation. The study was conducted between 2016 and 2019 across seven clinical sites located in three cities in South India. Detailed descriptions of the STRiDE cohort have been published previously [33]. For the present nested cohort study within the STRiDE cohort, 100 pregnant women with fasting glucose levels < 5.1 mmol/L (92 mg/dl) in early pregnancy were randomly selected with similar age and pre-pregnancy body mass index (BMI) to minimize the potential confounding effects of these variables. At 24–28 weeks of gestation (OGTT visit), all participants underwent a 75 g oral glucose tolerance test (OGTT) and were subsequently classified as having either normal glucose tolerance (NGT, *n* = 50) or gestational diabetes mellitus (GDM, *n* = 50) according to the International Association of Diabetes and Pregnancy Study Groups (IADPSG) criteria [34]. Metabolomic profiling was performed for all 100 participants at early pregnancy, and the overall study design is illustrated in Supplementary Fig. 1. We implemented a nested case–control sampling strategy within the STRiDE cohort to facilitate the matched analysis and control for key confounders. Fifty women who developed GDM at 24–28 weeks of gestation were identified, and for each case, one normoglycemic control was selected from the same cohort, matched on age and pre-pregnancy BMI (group matched) to yield a 1:1 matched set. This procedure led to equal numberof cases and controls by design.

A written informed consent was obtained from all participants. The study protocol was approved by the Institutional Ethics Committees of the National Institute of Mental Health and Neuro Sciences (NIMHANS), Bengaluru, and the Madras Diabetes Research Foundation (MDRF), Chennai, India.

### Polar sample preparation

The samples were extracted based on an extraction method reported by Evans et al. [35]. This method was uniformly used for all the samples and quality controls (QCs). The QC samples were prepared by combining equal volume from each individual sample within each category. Briefly, the plasma samples were thawed on ice prior to further processing, and the labelled internal standards (^13^C_6_ - Glucose andD_5_ - Glutamic acid) were added to 50 µL of the plasma samples and vortexed for 5 minutes. Subsequently, the sample deproteinization was performed by adding 450 µL of ice-cold methanol. The mixture was incubated for 1 h at -80 °C. The samples were centrifuged at 13,500 rpm for 15 minutes at 8 °C. The supernatant was collected and then transferred into a new sample tube.

### Non-polar sample preparation

The samples were extracted based on an extraction method reported by Cajka et al. [36]. This method was uniformly used for all the samples and QCs. The QC samples were prepared by combining equal volume from each individual sample within each category. The sample deproteinization was performed by adding 450 µL of ice-cold methanol containing Sphingomyelin (d18:0/17:0), Sphingosine (d17:1) and Ceramide (d18:0/17:0) standards. 1 mL of MTBE was added. Phase separation was induced by adding 250 µL of LC-MS grade water, followed by centrifugation at 14,000 rpm for 5 min at 4 °C. The upper organic phase was collected and dried under vacuum at 10 °C. Finally, the dried samples were reconstituted with ACN and IPA (2:1 v/v). 50 µL of each sample was added to the autosampler vial prior to sample analysis.

### UPLC-MS/MS analysis

Polar and non-polar analysis was performed on Ultra Performance Liquid Chromatography (UPLC) (Water^®^ ACQUITY I-Class), equipped with a binary solvent manager with online degasser, flow-through needle (FTN) sample manager, and column oven compartment. In polar metabolite analysis, Waters Acquity BEH HILIC column (50 mm*2.1 mm, 1.7 μm) was used for chromatography separation and column temperature was maintained at 45 °C. 1 µL of sample was injected for analysis. The polar analytes were separated via multistep gradient elution and flow rate. The mobile phase A was composed of 0.1% formic acid in water (v/v) with 10 mM ammonium acetate, and mobile phase B was 0.1% formic acid in acetonitrile: water (95:5) with10 mM ammonium acetate. The gradient conditions were; at 0 min-0.15mL, 10% (A); at 0.25 min-0.15 mL, 10% ( A); at 0.5 min-0.15 mL, 20% ( B); at 4.0 min-0.15 mL, 25% ( A); at 4.5 min-0.35 mL, 40% ( A); at 7.0 min-0.35 mL, 90% ( A); at 7.10 min-0.35 mL, 10% ( A); at 8.0 min-0.35 mL, 10% ( A); at 8.01 min-0.15 mL, 10% ( A); at 10.0 min-0.15 mL, 10% ( A).

For non-polar and polar lipid analysis, a Waters Acquity BEH Amide column (50 mm*2.1 mm, 1.7 μm) was used for chromatography separation, and the column temperature was maintained at 45 °C. 2 µL of the sample was injected for analysis. The lipids were separated via multi-step gradient elution. The mobile phase A was composed of 10 mM ammonium acetate in acetonitrile: water (95:5) and mobile phase B was 10 mM ammonium acetate in acetonitrile: water (95:50). Flow rate was set at 0.6 mL/min. The gradient conditions were at 0 min, 99.9% (A); at 1.0 min, 80% (A); at 2.0 min, 20% (B); at 2.50 min, 99.9% (A); at 4. min, 99.9% (A). For polar, non-polar and polar lipid analysis, the purge solvent was a mixture of 50% acetonitrile in water, while the needle wash was 80% acetonitrile in water.

The polar, nonpolar and polar lipid metabolite analysis was performed on Xevo TQ-XS tandem Mass Spectrometer (Waters Corporation) with an ESI ionization source in both positive and negative mode MRM. The source parameters for each analyte were optimized using standards and pooled samples. The MS source capillary was maintained for positive and negative ion mode at 3.0 kV, 2.5 kV respectively. The desolvation, cone, and collision gas were maintained at 1000 L/h, 150 L/h, and 0.13 mL/min, respectively, and the nebulizer gas was operated at 7.0 bar. UPLC-MS/MS analysis of the raw data was processed with MassLynx v4.2 and TargetLynx (Waters Corporation) for peak detection, alignment, and quantification. Internal standards were employed to maintain uniformity across runs, and pooled QC samples were incorporated throughout the study to assess technical variability and instrument performance.

### Statistical analysis

The metabolomic data comprised two datasets: Polar and Non-Polar Dataset. The Non-Polar dataset was further subdivided into positive ion mode and negative ion mode subsets. Initial quality assessment was conducted using relative standard deviation (RSD) values calculated fromQC samples. The following filtering criteria were applied: (a) Metabolite abundance with the RSD greater than 20% was replaced with missing values (“Null”). (b) Metabolites with an RSD of 20% or less were retained without modification. These adjustments were made to the averaged dataset using the RSD values as a reference. The same preprocessing procedure was consistently applied across all three datasets: polar, positive non-polar lipids, and negative polar lipids.

### Data cleaning and imputation

The metabolites inclusion workflow is presented in Supplementary Fig. 2. Out of 651 metabolites, 278 passed QC assessment. The RSD-filtered datasets were imported into Python for further processing. For handling missing data, we excluded metabolites with > 40% missing values, and for the remaining metabolites with ≤ 40% missing values, imputation was performed by replacing missing entries with half the minimum observed value for that metabolite. Boxplots were generated prior to and following data transformations to evaluate distributions and to identify outliers (Supplementary Figs. 3, 4 and 5). To stabilize variance and normalize distributions, all metabolite values were log-transformed plus one to handle infinity. To address potential batch effects, the pyComBat package was employed. Post-adjustment boxplots were generated to assess batch normalization both across batches and individual metabolites (Supplementary Fig. 3). Subsequently, data was standardized using the StanderScaler function from the scikit-learn package, which scales features to zero mean and unit variance. Principal Component Analysis (PCA) was performed on the standardized datasets to reduce dimensionality and identify key contributors to variance. Metabolites were ranked according to their loading scores on the principal components. Following preprocessing, the three datasets were merged using unique Batch IDs and Sample IDs to maintain alignment and prevent redundancy, metabolite full annotation is given in the Supplementary Table 1. For the characteristics of clinical variables: Continuous variables were summarized as mean ± standard deviation (SD), and categorical variables were presented as frequencies and percentages (n [%]). The Kolmogorov–Smirnov test was used to assess data normality, confirming the use of parametric tests due to the normal distribution of the variables. Comparisons of continuous variables between groups were performed using independent t-tests, while categorical variables were compared using the Chi-square test or Fisher’s exact test, as appropriate.

### Association and prediction analysis

Logistic regression analysis was conducted to examine the associations between individual metabolites and GDM. Odds ratios (ORs) were estimated for each metabolite by modeling GDM status as the dependent variable and metabolite levels as independent variables. A significance threshold of FDR adjusted *p* value < 0.05 was applied following correction for multiple testing. In addition, a Random Forest (RF) classification algorithm was employed to evaluate the predictive performance of metabolites for GDM. For the machine learning analysis, we used a stratified random partitioning approach. In each of ten iterations, the dataset was split into 70% training (n = 70) and 30% test (n = 30) subsets, stratified by case/control status. Within each training subset, we performed stratified 10-fold cross-validation for model tuning. Random forest hyperparameters were tuned using a grid search over mtry values and ntree values. Hyperparameter optimisation was performed exclusively within the training folds to prevent information leakage. A fixed random seed was applied to ensure reproducibility across iterations. Evaluation metrics included the area under the receiver operating characteristic curve (AUROC), F1 score, and overall accuracy, providing a comprehensive assessment of both sensitivity and specificity of the classification model. Feature importance scores were derived to identify metabolites contributing most to the prediction of GDM. To identify robust biomarker panels, a systematic approach involving ten iterations of random data partitioning into training and testing sets were employed, with reproducibility ensured through fixed random seeds. In each iteration, panels comprising 1 to 10 metabolites were constructed and evaluated for classification performance based on the AUC.

Model stability was assessed using bootstrap validation with 1000 resampling runs.

For each panel, performance metrics including AUC, sensitivity, specificity, and accuracy were aggregated, and their SDs were calculated. The most stable model within each panel size was defined as the one with the lowest SD. From these, the final biomarker panel was selected based on the highest AUC among the top models across all iterations. To assess whether the predictive performance of our machine learning model exceeded chance, we performed permutation testing. Outcome labels were randomly shuffled 1,000 times, and the model was refitted for each permutation using the same cross-validation and preprocessing procedures. The distribution of performance metrics (e.g., accuracy, AUROC) from the permuted datasets were compared with the performance of the original model. A model performance significantly higher than the permuted distribution indicates that the observed patterns are unlikely to have arisen by chance, supporting the robustness of the identified signals.

All analyses were executed using R (version 4.2.2), employing the “train” function from the caret package to implement the RF. The predictive performance of the logistic regression model was assessed by constructing AUROC and corresponding 95% confidence intervals (CI) reported as primary measures of model discrimination. All statistical analyses were performed using R software (version 4.3.2), and a two-tailed *p* value < 0.05 was considered statistically significant.

### Clinical model developments

Conventional risk predictors based on clinical accessibilitynullWe considered the clinically accessible maternal risk factors and blood-based metabolomics signatures associated with the development of GDM, utilizing data from the original STRiDE cohort of 2,115 pregnant women [33]. These factors were included in the nested GDM case-control subset as potential conventional risk predictors and comprised maternal age, waist-to-height ratio, family history of diabetes, and venous HbA1c. All selected predictors are routinely measured in clinical practice at relatively low cost.

**Fig. 1:**
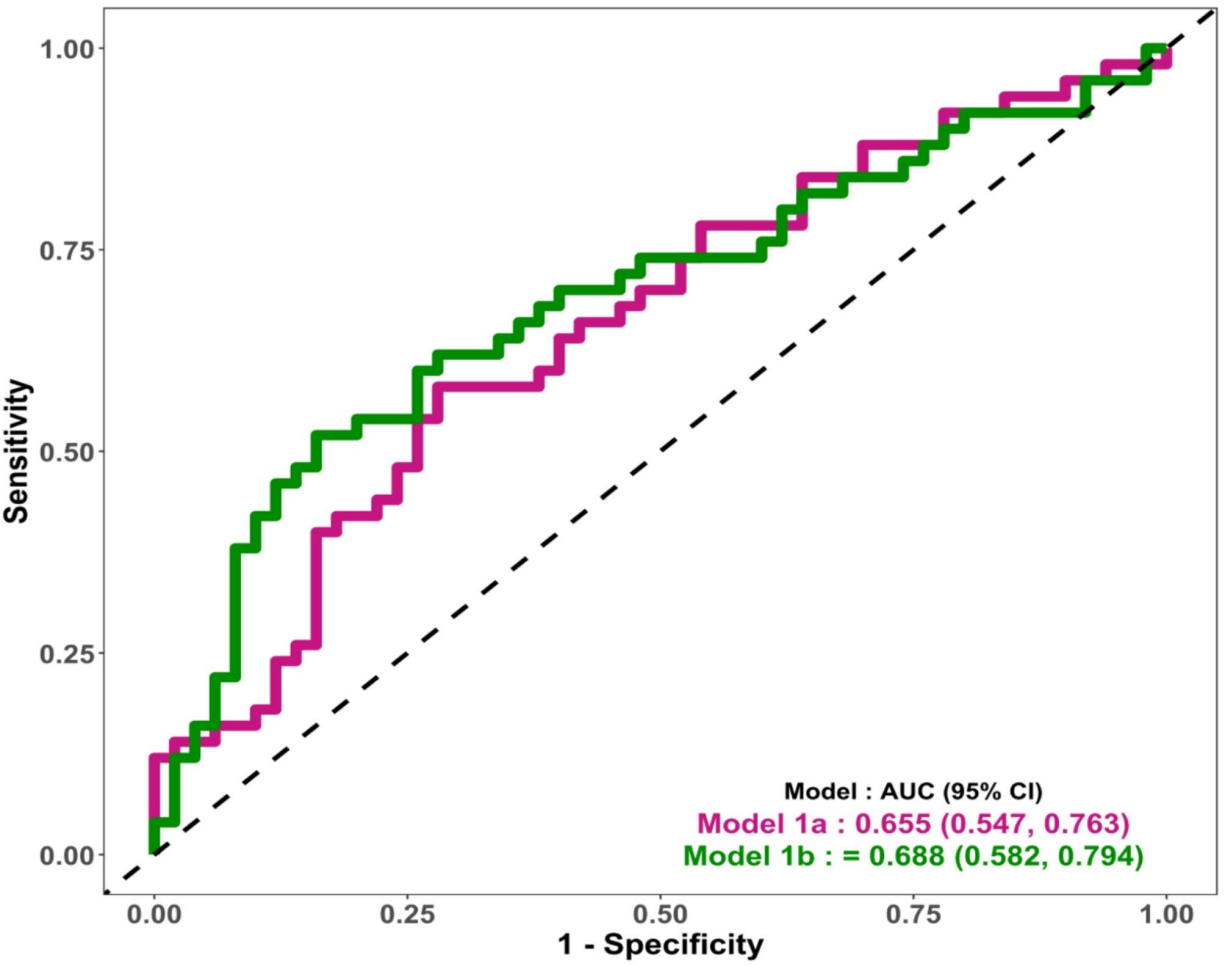
GDM prediction using conventional risk factors. Model 1a: Composite risk score (venous HbA1c, age, BMI, and family history of diabetes) for GDM prediction. 1b: Conventional risk predictors (age, waist-to-height ratio, family history of diabetes and venous HbA1c at early pregnancy) for GDM prediction

Initially, logistic regression models were constructed using maternal risk factors assessed during early pregnancy, with GDM status at the OGTT visit as the outcome. In Model 1a, maternal age, pre-pregnancy BMI, family history of diabetes, and venous HbA1c were included as covariates. Model 1b tested substituting waist-to-height ratio for pre-pregnancy BMI to evaluate the potential influence of central adiposity while adjusting for the same covariates from Model 1a (Fig. 1).

### Metabolomic signature predictors

This includes novel metabolomic signatures identified through mass spectrometry profiling. These biomarkers are currently not available for routine clinical use and require specialized laboratory facilities for measurement. We further integrated conventional maternal risk factors with novel metabolomic signatures to develop a clinically applicable and comprehensive model for GDM risk prediction.

### Metabolic pathway enrichment analysis

Metabolic pathway enrichment analysis was performed using KEGG compound identifiers (e.g., C00031, C00186). Metabolite-to-pathway associations were retrieved via the KEGG REST API, and a custom enrichment approach was implemented in R specifically for the human analysis. For each KEGG pathway, the overlap between input metabolites and known pathway-associated metabolites were evaluated using Fisher’s exact test. *p* values were adjusted for multiple testing using the Benjamini-Hochberg method to control the false discovery rate (FDR). Pathways with an FDR-adjusted *p* value < 0.05 were considered significantly enriched. Pathway annotations were retrieved using the keggList function to associate KEGG map IDs with pathway names.

## Results

### Characteristics of the study participants

Baseline and follow-up characteristics of the study population are shown in Table 1. The nested case-control samples included 100 women (50 GDM cases and 50 NGT controls), drawn from the larger STRiDE cohort (*n* = 2115). In early pregnancy (< 16 weeks), there were no significant differences in mean age or gestational age at inclusion between GDM and NGT groups. The average age was approximately 26–27 years across all groups, with recruitment occurring at a mean gestational age of 10 weeks. Anthropometric measures showed a trend towards higher pre-pregnancy weight, BMI and waist circumference in the GDM group compared to the NGT group. Blood pressure measurements were comparable between groups at both visits. Fasting plasma glucose and HbA1c levels during early pregnancy were similar in both groups (FPG: 83 mg/dL; HbA1c: 5.02%), indicating that glycemic profiles were largely indistinguishable prior to the development of GDM. A higher proportion of GDM participants reported a family history of T2DM (54%) compared to NGT participants (40%). Additionally, a greater number of GDM cases belonged to the lower socioeconomic class (20% vs. 8%), suggesting a potential role for familial and socio-demographic factors in GDM risk. At the OGTT visit (24–28 weeks), a significant difference emerged in glucose regulation. Fasting, 1-hour, and 2-hour plasma glucose levels were higher in the GDM group compared to the NGT group (*p* < 0.01 for all). Venous HbA1c was also modest but significantly higher in GDM participants (4.92 ± 0.3%) compared to controls (4.76 ± 0.3%, *p* < 0.05). These findings highlight that while early pregnancy glycemic markers were similar across groups, clear metabolic differences emerged by mid-pregnancy, reinforcing the importance of dynamic glucose testing (i.e., OGTT) for timely identification of GDM.

**Table 1:** Baseline characteristics at different visits of pregnancy

Variables	All STRiDE participants (*n* = 2115)	All (Nested case -control) participants (*n* = 100)	NGT (*n* = 50)	GDM (*n* = 50)
*Early pregnancy (< 16 weeks)*				
Age (years)	27 ± 4.1	26 ± 2.5	26 ± 2.4	27 ± 2.6
Gestational Age (weeks) at inclusion	10.5 ± 3	10.2 ± 3	10 ± 3.1	10.5 ± 3.1
Pre-pregnancy weight (kg)	59.2 ± 12.6	55.8 ± 8.1	55.3 ± 7.6	56.4 ± 8.6
Body mass index (kg/m^2^)	24.2 ± 4.6	23.3 ± 2.5	22.8 ± 2.1	23.8 ± 2.9
Waist Circumference (cm)	86 ± 10	83 ± 8	82 ± 7.5	85 ± 8.2
Systolic Blood Pressure (mm Hg)	102 ± 10	102 ± 10	101 ± 10.4	104 ± 10
Diastolic Blood Pressure (mm Hg)	69 ± 9	67 ± 8	66 ± 8	68 ± 7.5
Fasting plasma glucose (mg/dl)	83 ± 5.2	83 ± 4.5	83 ± 5.2	83 ± 3.9
HbA_1c_ (Venous. %)	5.08 ± 0·3	5.02 ± 0.3	5.02 ± 0.3	5.02 ± 0.3
Family history of type 2 diabetes n [%]	860 (40.7)	47 (47)	20 (40)	27 (54)
Socio Economic Status (SES) n (%)				
Lower Class	499 (23.6)	14 (14)	4 (8)	10 (20)
Middle Class	1177 (55.7)	60 (60)	32 (64)	28 (56)
Upper Class	439 (20.8)	26 (26)	14 (28)	12 (24)
Nulliparous n (%)	1291 (61)	84 (84)	50 (100)	34 (68)*
*OGTT Visit (24–28 weeks)*				
Systolic Blood Pressure (mm Hg)	102 ± 10.5	101 ± 9.5	100 ± 9.4	102 ± 9.7
Diastolic Blood Pressure (mm Hg)	68.8 ± 9.4	66 ± 7.25	65.6 ± 7.9	66.5 ± 6.6
OGTT Fasting plasma glucose (mg/dl)	81.5 ± 8.1	82.7 ± 6.1	80.5 ± 5.7	85 ± 6.5**
OGTT 1 h Venous plasma glucose (mg/dl)	137 ± 30.8	154 ± 26	129 ± 23.3	179 ± 28.7**
OGTT 2 h Venous plasma glucose (mg/dl)	118 ± 26.1	130 ± 22.7	112 ± 16.8	148 ± 28.6**
HbA_1c_ (Venous. %)	5.08 ± 0.3	4.84 ± 0.3	4.76 ± 0.3	4.92 ± 0.3*

Data presented as mean ± SD. *p < 0.01, **p < 0.001 compared to NGT. OGTT—Oral Glucose Tolerance Test

### Metabolomic signatures in association with GDM

**Fig. 2:**
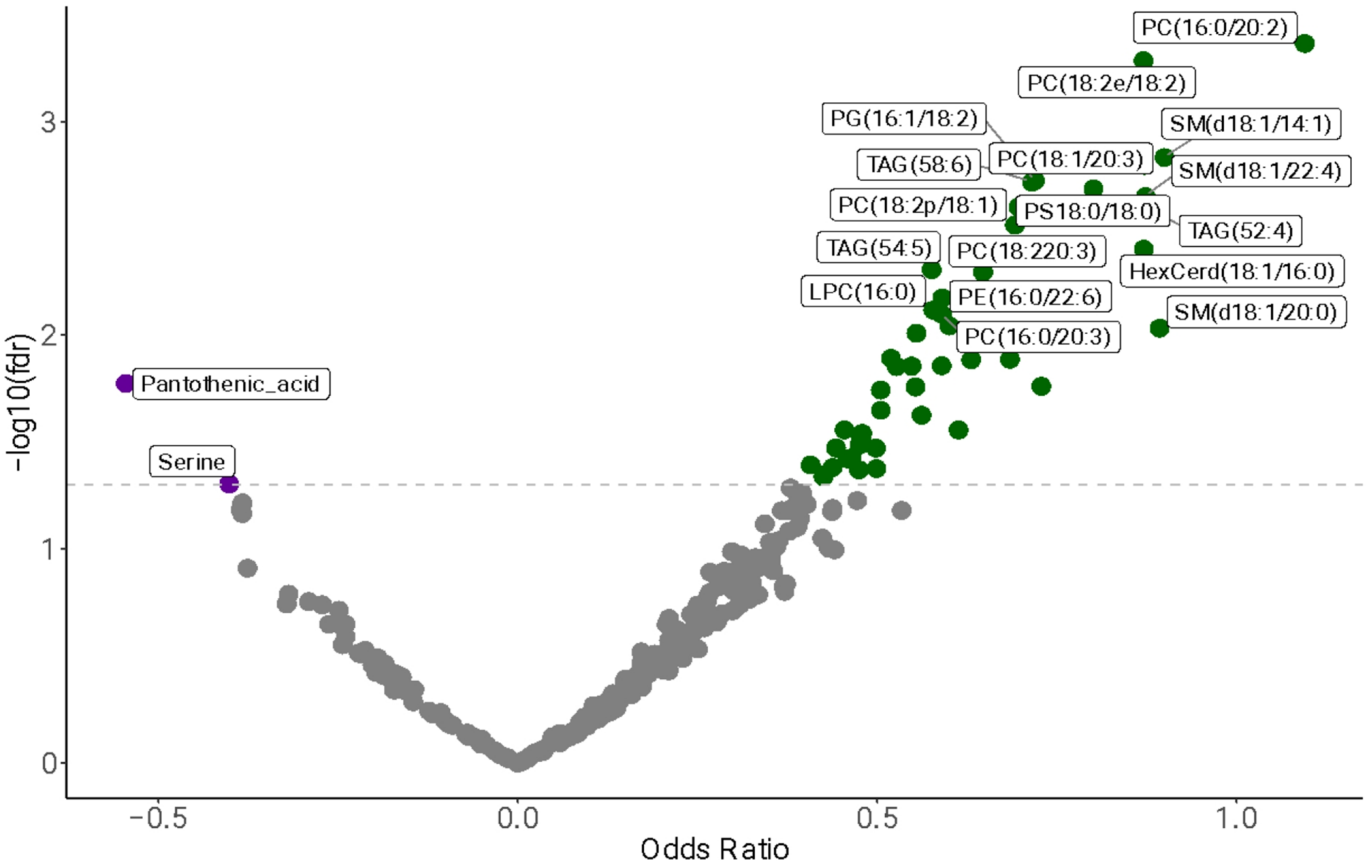
Volcano plot showing the association of metabolites with GDM. Each point represents a metabolite, plotted according to the odds ratio (OR) on the x-axis and the –log_10_ of the FDR-adjusted *p* value on the y-axis from logistic regression analysis. Metabolites with OR > 1 and significant *p* values (above the horizontal threshold line) are considered positively associated with GDM and are colored in green; those with OR < 1 and significant *p* values are negatively associated and shown in blue. Non-significant metabolites are shown in gray. The horizontal dashed line denotes the significance threshold (FDR-adjusted *p* values < 0.05)

A total of 49 metabolites were found to be significantly associated with GDM based on logistic regression analysis (Fig. 2). Notably, lipid classes such as phosphatidylcholines (PC), sphingomyelins (SM), and triacylglycerols (TAG) were predominantly positively associated with GDM. Among the positively associated metabolites, PC(16:0/20:2) (OR = 1.09; 95% CI: [0.54, 1.76]; FDR-adjusted *p* = 4.28 × 10^−4^), PC(18:2/18:2) (OR = 0.87; 95% CI: [0.41, 1.33]; FDR-adjusted *p* = 5.1 × 10^−4^), and SM(d18:1/14:1) (OR = 0.89; 95% CI: [0.40, 1.52]; FDR-adjusted *p* value = 0.001) demonstrated the strongest associations. Pantothenic acid (Estimate = − 0.54; 95% CI: [–1.02, − 0.12]; FDR-adjusted *p* = 0.016) and Serine (OR = − 0.40; 95% CI: [–0.82, − 0.017]; FDR-adjusted *p* = 0.049) were negatively associated with GDM. Full statistical details for the significant 49 metabolites are provided in Table 2, while comprehensive results for all metabolites are available in Supplementary Table 2.

**Table 2:** Summary of logistic regression analysis evaluating the association between individual metabolites and GDM

Metabolite	Estimate	CI 2.5%	CI 97.5%	SE	*p*-value	FDR-adjusted *p*-values
PC(16:0/20:2)	1.09	0.54	1.76	0.31	4.28E−04	4.28E−04
PC(18:2e/18:2)	0.87	0.42	1.40	0.25	5.15E−04	5.15E−04
SM(d18:1/14:1)	0.90	0.40	1.53	0.28	1.46E−03	1.46E−03
PC(18:1/20:3)	0.87	0.37	1.46	0.28	1.59E−03	1.59E−03
PG(16:1/18:2)	0.72	0.29	1.20	0.23	1.88E−03	1.88E−03
TAG(58:6)	0.71	0.30	1.21	0.23	1.90E−03	1.90E−03
PC(16:0/22:6)	0.80	0.33	1.36	0.26	2.05E−03	2.05E−03
SM(d18:1/22:4)	0.87	0.38	1.50	0.29	2.23E−03	2.23E−03
PC(18:2p/18:1)	0.70	0.28	1.19	0.23	2.50E−03	2.50E−03
TAG(52:4)	0.89	0.38	1.55	0.29	2.65E−03	2.65E−03
PS(18:0/18:0)	0.69	0.25	1.17	0.23	3.03E−03	3.03E−03
HexCerd(18:1/16:0)	0.87	0.35	1.55	0.30	3.92E−03	3.92E−03
TAG(54:5)	0.58	0.19	1.00	0.20	4.90E−03	4.90E−03
PC(18:220:3)	0.65	0.22	1.13	0.23	5.02E−03	5.02E−03
PE(16:0/22:6)	0.59	0.17	1.03	0.22	6.63E−03	6.63E−03
LPC(16:0)	0.58	0.17	1.02	0.22	7.56E−03	7.56E−03
PC(16:0/20:3)	0.59	0.18	1.06	0.22	7.91E−03	7.91E−03
PC(18:2e/18:3)	0.60	0.19	1.09	0.23	8.96E−03	8.96E−03
SM(d18:1/20:0)	0.89	0.34	1.71	0.34	9.19E−03	9.19E−03
PE(22:322:6)	0.55	0.14	0.99	0.21	9.70E−03	9.70E−03
PC(18:1/e18:3)	0.52	0.14	0.96	0.21	1.27E−02	1.27E−02
PC(18:0/p18:0)	0.68	0.21	1.31	0.28	1.29E−02	1.29E−02
TAG(56:4)	0.63	0.17	1.18	0.25	1.29E−02	1.29E−02
PC(16:0/22:4)	0.59	0.15	1.10	0.24	1.38E−02	1.38E−02
PE(18:0/20:2)	0.55	0.13	1.01	0.22	1.38E−02	1.38E−02
SM(d18:1/16:1)	0.53	0.14	0.98	0.21	1.39E−02	1.39E−02
Pantothenic_acid	-0.55	-1.02	-0.12	0.23	1.67E−02	1.67E−02
SM(d18:1/22:1)	0.73	0.24	1.45	0.31	1.72E−02	1.72E−02
PC(16:0/p20:4)	0.55	0.14	1.05	0.23	1.73E−02	1.73E−02
SM(d18:1/18:2)	0.50	0.11	0.95	0.21	1.79E−02	1.79E−02
PC(14:0/16:0)	0.50	0.08	0.95	0.22	2.22E−02	2.22E−02
PC(18:0/20:2)	0.56	0.12	1.09	0.25	2.35E−02	2.35E−02
PE(18:0/22:6)	0.45	0.06	0.87	0.21	2.75E−02	2.75E−02
DAG(16:1/16:1)	0.61	0.13	1.24	0.28	2.75E−02	2.75E−02
PE(18:0/18:1)	0.48	0.06	0.92	0.22	2.85E−02	2.85E−02
LPC(20:3)	0.48	0.08	0.95	0.22	3.03E−02	3.03E−02
TAG(52:5)	0.47	0.05	0.93	0.22	3.24E−02	3.24E−02
PC(18:1/e20:4)	0.44	0.06	0.88	0.21	3.34E−02	3.34E−02
PE(18:0/20:4)	0.50	0.06	0.99	0.23	3.34E−02	3.34E−02
PC(18:1/20:2)	0.46	0.04	0.92	0.22	3.72E−02	3.72E−02
TAG(50:2)	0.46	0.04	0.91	0.22	3.76E−02	3.76E−02
PC(16:0/e18:2)	0.41	0.05	0.85	0.20	4.01E−02	4.01E−02
PG(16:1/18:1)	0.44	0.04	0.89	0.21	4.12E−02	4.12E−02
PE(16:0/18:2)	0.50	0.04	1.01	0.24	4.17E−02	4.17E−02
PC(16:1/18:1)	0.47	0.05	0.98	0.23	4.23E−02	4.23E−02
PC(16:0/22:2)	0.43	0.02	0.85	0.21	4.44E−02	4.44E−02
PC(16:0/18:3)	0.42	0.02	0.86	0.21	4.55E−02	4.55E−02
Serine	-0.40	-0.83	-0.02	0.20	4.92E−02	4.92E−02

The table includes the estimated regression coefficient (Estimate), standard error (SE), 95% confidence interval bounds (CI 2.5% and CI 97.5%), nominal *p* values, and false discovery rate FDR adjusted *p* values for multiple testing correction.

### Metabolomic association with other clinical variables

**Fig. 3:**
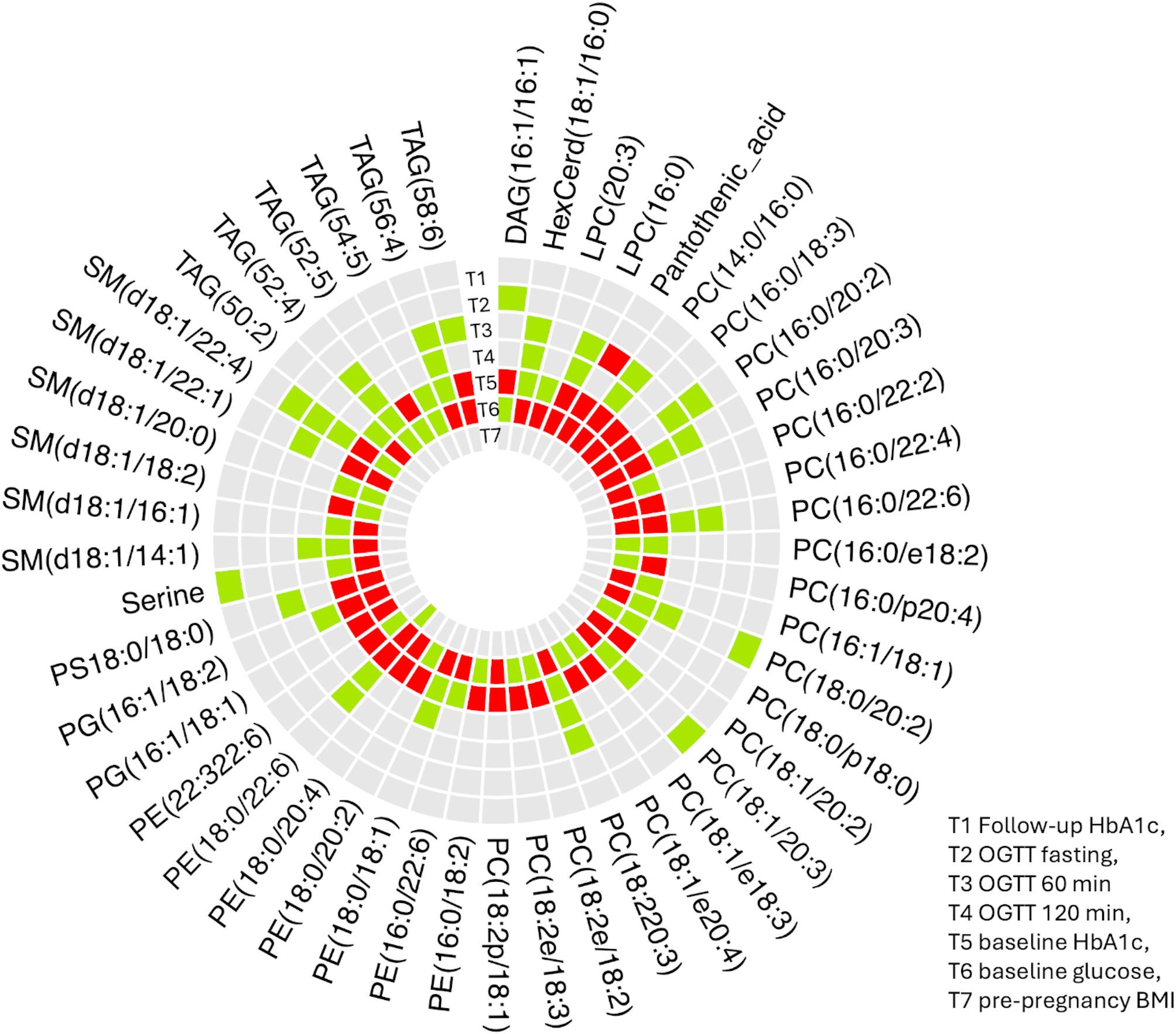
Circos heatmap plot illustrating associations between baseline metabolites and clinical variables related to glycemic outcomes. The outermost circle displays metabolite names. Inner rings represent clinical variables including follow-up HbA1c, OGTT fasting, 60 min and 120 min, baseline HbA1c, baseline glucose, and pre-pregnancy BMI. Colored bars within each ring indicate the direction of association between each metabolite and the corresponding variable (green: upregulated; red: downregulated; grey: no change)

To investigate temporal changes in metabolite signatures across pregnancy, we generated a circular heatmap (Circos plot) illustrating differentially abundant metabolites across seven study timepoints: follow-up HbA1c (T1), oral glucose tolerance test phases (T2–T4), baseline HbA1c (T5), baseline glucose (T6) and pre-pregnancy BMI (T7). Metabolites, including PCs, phosphatidylethanolamines (PEs), SMs, TAGs, and other small molecules such as serine and pantothenic acid, were mapped based on their relative abundance and statistical significance at each timepoint. The plot revealed dynamic and time-specific shifts in lipid profiles. Several PCs (e.g., PC(16:0/22:6), PC(18:2e/18:3) and PEs (e.g., PE(18:0p/22:6) were significantly upregulated during OGTT phases (T2–T4) and baseline (T5–T6), suggesting enhanced lipid remodeling or signaling activity during glucose challenge. Conversely, certain SMs (e.g., SM(d18:1/22:1) and amino acids like serine were downregulated at early time points, particularly in the pre-pregnancy group (T7), indicating potential preclinical metabolic alterations (Fig. 3).

### Predictive analysis of metabolite signatures for GDM using machine learning

For the development of a conventional risk prediction model for GDM, we initially built a model using the maternal risk factors and blood-based biomarkers, linked to the development of GDM (AUC: 0.65) from the original cohort of 2,115 pregnant women (Fig. 1: Model 1a). The nested GDM case-control subset included the same factors and other potential conventional risk predictors. The predictors considered were age, waist-to-height ratio, family history of diabetes, and venous HbA1c, all routinely assessed at relatively affordable clinical costs. This prediction model had an AUC of 0.688 for GDM (Fig. 1: Model 1b).

For the development of the metabolomic signature panel and to evaluate its performance for early GDM prediction, we applied the RF machine learning method and bootstrap validation with 1000 repetitions, respectively, on a metabolomics dataset of 49 metabolites that were significantly associated. The RF method identified a panel of eight metabolites (PC(18:1/20:3), PC(16:0e/18:2), TAG(50:2), Serine, PE(18:0/20:4), HexCer(d18:1/16:0), SM(d18:1/14:1), and PS(18:0/18:0) with the best prediction performance for the GDM. During bootstrap validation, the panel maintained robust performance, yielding a mean AUC of 0.83, a mean sensitivity of 0.73 and a specificity of 0.75. Table 3 summarises the performance metrics for the selected 8 metabolite panel. Predictions for all other metabolites panels are given in the Supplementary Table 3. The mean levels of eight metabolites are presented as a violin plot in the Supplementary Fig. 6. In the iterative train/test splits, the eight-metabolite panel achieved a mean test-set AUC of 0.98. However, when assessed by 1,000-repeat bootstrap resampling, the mean AUC was 0.83 (95% CI: 0.77–0.89; SD: 0.06). The bootstrap estimate provides a more conservative measure of performance and reflects the variability expected in independent samples. The discrepancy between the single split test AUC and the bootstrap AUC likely reflects sampling variability due to the modest sample size. Further, permutation testing demonstrated that the model’s predictive performance was significantly higher than expected by chance (*p* < 0.001) (Supplementary Fig. 7, indicating that the identified metabolomic/lipidomic patterns are robust despite the limited sample size.

**Table 3:** Performance of the 8-Metabolite panel selected by random forest for GDM prediction

Method	Dataset	AUC	Sensitivity	Specificity	F1 Score	Accuracy
Random Forest (8 metabolites)	Test Set	0.98	1	0.56	0.82	0.78
	Bootstrap (Mean)	0.83	0.73	0.75	—	0.74

**Fig. 4:**
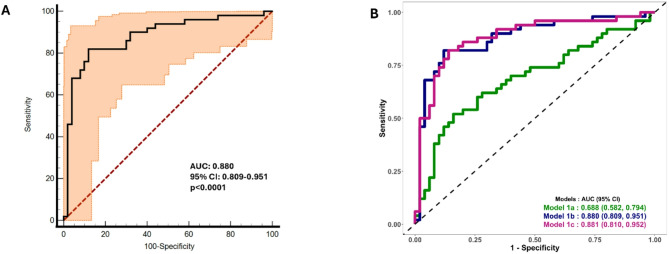
ROC analysis of the 8 metabolomic markers and conventional risk factors at early pregnancy. **a** ROC showing the novel 8 metabolomic signatures as metabolomic biomarker predictors of GDM mean in black curve and CI in orange area. **b** ROC curves and AUC of different prediction models. Model 1a: Conventional risk predictors (age, waist-to-height ratio, family history of diabetes and HbA1c), Model 1b: Novel metabolomic biomarker panel of 8 markers at early pregnancy. Model 1c: Full GDM prediction model - integrated clinically accessible maternal risk factors (conventional risk predictors) with novel metabolomic panel of 8 signatures (1a & 1b combined)

After obtaining the best panel of 8 metabolites through building step-by-ML approaches and bootstrap validation, further logistic regression models were constructed to deepen the understanding of these metabolites and their association with other maternal risk factors linked to GDM prediction. ROC analysis demonstrated a robust AUC of 0.880 (95% CI: 0.809– 0.951) with a sensitivity of 82% and a specificity of 88% (Fig. 4a).

To develop a clinically efficient comprehensive GDM prediction model, we integrated clinically accessible maternal risk factors (conventional risk predictors) with novel 8 metabolomics signatures. Interestingly, the addition of the conventional risk predictors has slightly increased the AUC of the predicted GDM risk to 0.881, suggesting that the full GDM prediction model at early pregnancy improved the discrimination even after adjusting for the potential maternal risk factors (Fig. 4b: Model 1c). Parity, a known risk factor for GDM, was summarized in Table 1. As a sensitivity analysis, we have adjusted our model for parity, and no significant differences were observed in the associations of the eight metabolites with GDM status.

### Pathway enrichment analysis

**Fig. 5:**
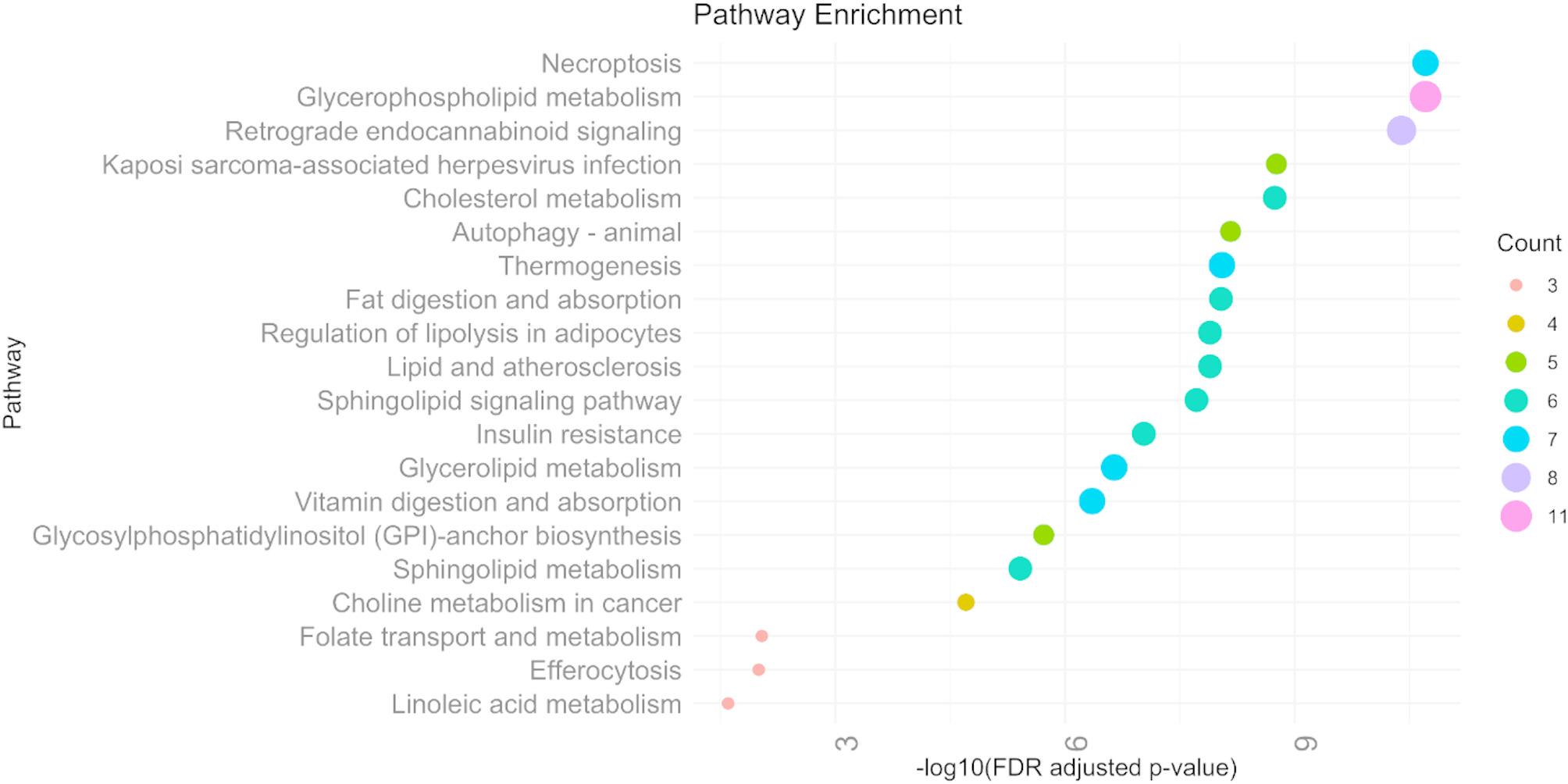
Dot plot showing the top 10 enriched KEGG pathways based on significantly altered metabolites. The y-axis represents enriched pathways, and the x-axis shows –log_10_ (FDR adjusted *p* value) from a hypergeometric test. Dot sizes correspond to the number (as a count) of metabolites associated with each pathway

Pathway enrichment analysis identified several significantly enriched metabolic and signaling pathways (FDR-adjusted *p* values < 0.05) associated with the studied condition (Fig. 5). Notably, lipid-related pathways were highly represented, including Glycerophospholipid metabolism (FDR-adjusted *p* values = 1.96 × 10^−11^), Glycerolipid metabolism (FDR-adjusted *p* values = 2.28 × 10^−7^), Sphingolipid metabolism (FDR-adjusted *p* values = 3.85 × 10^−6^), and Cholesterol metabolism (FDR-adjusted *p* values = 1.82 × 10^−9^).

In addition, signaling and regulatory pathways such as Sphingolipid signaling (FDR-adjusted *p* values = 1.92 × 10^−8^), Autophagy (FDR-adjusted *p* values = 6.88 × 10^−9^), Necroptosis (FDR-adjusted *p* values = 1.96 × 10^−11^), and Retrograde endocannabinoid signaling (FDR-adjusted *p* values = 4.02 × 10^−11^) were significantly enriched. Other relevant findings included pathways involved in insulin resistance, fat/vitamin digestion and absorption, regulation of lipolysis, and folate metabolism. These results underscore the involvement of lipid metabolism, cell death pathways, and metabolic regulation in the underlying GDM pathophysiology.

## Discussion

Recent studies have consistently highlighted disruptions in lipid metabolism [28, 29],amino acid turnover [37], and energy homeostasis [38] in the pathogenesis of GDM. In this context, using high-throughput UPLC-MS/MS within the nested STRiDE cohort, we identified 49 metabolites, measured during early pregnancy, significantly associated with later GDM development. The results emphasise the contribution of lipid metabolism, cell death mechanisms, and lipid metabolic regulation to the underlying pathophysiology of GDM. Importantly, machine learning models revealed that a panel of eight metabolites could predict GDM with high accuracy, underscoring the potential clinical utility of metabolomics in early risk stratification. Traditional clinical risk factors alone provided the predictive power (AUC = 0.688), their integration with the metabolomic panel robustly improved prediction (AUC = 0.881), reinforcing the added value of molecular biomarkers in refining risk assessment. Findings from this metabolomic study in an Indian pregnancy cohort highlight the potential of early pregnancy novel metabolomic biomarkers, and this is the first comprehensive study to report such associations in early GDM prediction.

Studies by Zhao et al. and Scholtens et al., have demonstrated that metabolic derangements precede hyperglycemia, supporting the rationale for early screening using molecular signatures [17, 18]. Other studies, such as those by Guasch-Ferré et al., have integrated metabolomics with clinical risk factors to enhance predictive models, showing that a combination of metabolite panels and maternal characteristics can significantly improve early diagnostic accuracy compared to traditional screening alone [39]. Our findings corroborate and extend previous studies that identified early-pregnancy lipid perturbations associated with GDM. Like prior reports from Zhao et al., Scholtens et al. and Liu et al., we observed alterations in glycerophospholipids and sphingolipids. The novelty of the present study lies in its focus on an Indian population, among whom GDM prevalence is high, and in the combined profiling of polar and non-polar metabolites within a single analytical framework. The eight-metabolite panel we identified is therefore best viewed as a population-specific signature for an Indian population [40], that aligns with broader evidence linking early lipid metabolism to GDM risk. The eight metabolites identified by the random forest algorithm were PC(18:1/20:3), PC(16:0e/18:2), TAG(50:2), Serine, PE(18:0/20:4), HexCer(d18:1/16:0), SM(d18:1/14:1), and PS(18:0/18:0), represent diverse biochemical classes, including glycerophospholipids, sphingolipids, triacylglycerols, and amino acid derivatives. These classes of metabolites are central to GDM pathogenesis, such as insulin resistance [41] lipid imbalance [42], and mitochondrial dysfunction [43, 44].

PCs, both diacyl and ether-linked, were significantly elevated in women who later developed GDM. These findings align with previous studies that reported higher PCs in GDM and T2DM cohorts, such as those by Liu et al. and Zhao et al. [17, 29]. PC(18:1/20:3) may reflect enhanced hepatic lipid synthesis or impaired lipoprotein metabolism [45], both of which are common in insulin-resistant states. The ether-linked PC(16:0e/18:2) also signals oxidative stress and disrupted peroxisomal function, consistent with inflammatory activation observed in early GDM [46–48].

Triacylglycerols, such as TAG(50:2), are classical indicators of insulin resistance and dyslipidemia. Elevated TAG levels may indicate hepatic steatosis or impaired lipid clearance mechanisms, which are frequently observed in obese and GDM pregnancies [19, 39]. These results further validate the role of altered lipid storage and mobilization as early drivers of metabolic dysfunction in pregnancy.

The L-serine metabolism has shown to be altered in type 1, type 2, and gestational diabetes mellitus. Holem et al. [49], study suggests that L-serine supplementation can improve glucose homeostasis and mitochondrial function, indicating its potential therapeutic role in diabetes management. Ethanolamine is a precursor for PE, a mitochondrial membrane lipid essential for bioenergetics and insulin sensitivity [50]. Elevated PE(18:0/20:4), containing arachidonic acid, suggests a link to pro-inflammatory eicosanoid production and mitochondrial stress [51–53]. Barranco-Altirriba et al. [54] and Chang et al. [55] have highlighted the association of altered PE species with metabolic diseases, supporting our findings.

Hexosylceramides, a subset of sphingolipids, were increasingly recognized for their role in insulin resistance and β-cell dysfunction [56]. The strong positive association of HexCer(d18:1/16:0) with GDM from our study mirrors the findings by Wittenbecher et al., who demonstrated their predictive role for T2DM [57]. SMs modulate membrane microdomain structure, and their dysregulation may disrupt insulin receptor localization, thereby contributing to insulin resistance [58]. Beyond GDM, SMs are known to be associated with T2DM and mediating the effect of BMI on T2DM [59, 60]. We observed that SM(d18:1/14:1), were elevated in the GDM group, consistent with previous metabolomic studies in pregnant and non-pregnant populations [61]. Phosphatidylserine, though less studied in the context of GDM, plays a key role in cell signaling, apoptosis, and membrane structure [62]. Lappas et al. further supported these findings by showing that cholesteryl ester species, alkenyl phosphatidylethanolamines, and phosphatidylserine species were most strongly associated with the risk of developing T2DM after a GDM pregnancy [28]. Its elevation in GDM pregnancies may reflect altered placental membrane integrity or increased apoptotic signaling both potential contributors to impaired glucose homeostasis. Zhao et al., identified similar trends in related glycerophospholipid classes [17]. In this context, the pathway enrichment analysis in the present study revealed key pathways, including glycerophospholipid and sphingolipid metabolism, cholesterol metabolism, insulin resistance, necroptosis, and autophagy. These findings highlight the central role of lipid-related and signaling pathways in the GDM studies. Our findings serve as proof of concept and may inform future mechanistic studies exploring the potential role of these metabolomic signatures in the development of GDM across different ethnic groups. Additionally, the identified metabolomic panel may prove valuable for screening high-risk individuals. However, further validation in larger, independent cohorts and different ethnicities is needed to confirm these results.

The strengths of this study include that metabolomics results were drawn from a well-characterized, a nested case-control design embedded within a large, diverse cohort, enhancing internal validity. A high-resolution mass spectrometry capturing both polar and non-polar metabolite classes, enabling a broad metabolic landscape and rigorous statistical and machine learning validation strategies, including bootstrapping and cross-validation, increased robustness and minimised overfitting. Finally, our study focused on a high-risk population, Indian women, who exhibit higher GDM susceptibility and are underrepresented in metabolomic studies, enhancing the relevance and novelty of the findings.

This study has important limitations. First, the sample size of 100 women (50 GDM, 50 controls) is relatively modest. Although the nested case–control design strengthens internal validity, it also heightens the risk of overfitting in the machine learning analyses. Nevertheless, the statistical power estimation study by Blaise et al. [63] supports the adequacy of our sample size and validates the benchmark employed for the machine learning approach. Second, although we employed both stratified cross-validation and extensive bootstrap resampling to estimate performance, these are internal validation techniques. We did not validate the eight-metabolite panel in an independent external cohort, and therefore its clinical utility and generalisability remain speculative. Prospective validation in larger, geographically and ethnically diverse cohorts, ideally using targeted assays for the candidate metabolites, will be required before the panel can be considered for clinical translation. Third, our matching accounted for age and BMI but did not explicitly match on parity, although parity status was summarised and found not to differ materially between groups. Finally, our findings are derived from a single population and should be interpreted as exploratory. Despite stated limitations, the strength includes that this study was designed as an initial, hypothesis-generating nested case-control analysis within the large STRiDE cohort. The nested design enabled strict matching on key confounders and high-quality, early-pregnancy biosamples, which were profiled on the same UPLC-MS/MS platform, thereby reducing technical heterogeneity. These features enhance internal validity and render the identified metabolite classes biologically plausible (lipids, sphingolipids, phosphatidylcholines), consistent with prior literature. We performed extensive internal validation and complemented it with permutation testing, which confirmed that the model’s predictive performance was significantly greater than expected by chance. These results indicate that the identified metabolomic signatures are robust despite the modest sample size. We acknowledge that external validation is currently not feasible due to the lack of pregnancy focused cohorts with comparable metabolomics data in Indian populations, underscoring the novelty and unique contribution of our study. By generating high-resolution metabolomic profiles in this cohort, we provide a valuable metabolomics dataset that can serve as a foundation for future larger studies and for the upscaling of metabolomics research in this population. Moreover, our findings reinforce and extend earlier metabolomics studies. Prior studies by Scholtens et al. [18] and Zhao et al. [17] identified similar metabolic disruptions as an individual metabolite. Interestingly, our study is the first to identify an integrated 8-metabolite panel representing meaningful biology signals even with a modest sample size, in an Indian cohort, a population characterized by a high prevalence of GDM and distinct metabolic phenotypes.

## Conclusion

In summary, our study provides novel insights into early metabolic alterations associated with GDM in Indian women. An eight-metabolite panel identified through affordable MS-based profiling offers excellent predictive performance and reflects core biological processes implicated in GDM pathogenesis. These findings support the use of metabolomics as a powerful tool for early GDM screening and underscore the need for external validation and translational efforts to integrate these markers into clinical practice.

## Supplementary Information

Below is the link to the electronic supplementary material.

Supplementary Material 1: Table S1: Complete List of Metabolites with Annotations (Post-Preprocessing and Merging). Table S2: Details of all metabolites associated with GDM. Table S3: Prediction Performance Metrics of All Metabolite Panels for GDM. Figure S1: Study design. Figure S2: Metabolomics data QC work flow. Figure S3: Non-Polar Negative Data - Standardized. Boxplot of all the non-polar negative metabolites on x-axis and adjusted for the batch effect and scaled metabolite frequencies on y-axis. Figure S4: Non-Polar Positive Data - Standardized. Boxplot of all the non-polar positive metabolites on x-axis and adjusted for the batch effect and scaled metabolite frequencies on y-axis. Figure S5: Polar Positive Data - Standardized. Boxplot of all the polar metabolites on x-axis and adjusted for the batch effect and scaled metabolite frequencies on y-axis. Figure S6: A violin plot representing metabolites distribution across normal and GDM. Figure S7: Permutation test distribution of AUC.

## Data Availability

No datasets were generated or analysed during the current study.
